# Effect of Vaccination Time Intervals on SARS-COV-2 Omicron Variant Strain Infection in Guangzhou: A Real-World Matched Case–Control Study

**DOI:** 10.3390/vaccines10111855

**Published:** 2022-11-01

**Authors:** Yufen Li, Tong Guo, Jiayi Zhong, Chuanjun Fang, Husheng Xiong, Zengyun Hu, Yajuan Zhu, Jinlin Tan, Shuang Liu, Qinlong Jing, Dingmei Zhang

**Affiliations:** 1 Department of Epidemiology, School of Public Health, Sun Yat-Sen University, Guangzhou 510080, China; 2 Guangzhou Center for Disease Control and Prevention, Guangzhou 510440, China; 3 Guangzhou Center for Disease Control and Prevention, Institute of Public Health, Guangzhou Medical University, Guangzhou 510180, China; 4 Department of Public Health and Preventive Medicine, School of Medicine, Jinan University, Guangzhou 510632, China; 5 State Key Laboratory of desert and Oasis Ecology, Xinjiang Institute of Ecology and Geography, Chinese Academy of Sciences, Urumqi 830011, China; 6 NMPA Key Laboratory for Quality Monitoring and Evaluation of Vaccines and Biological Products, Guangzhou 510080, China

**Keywords:** breakthrough infection, COVID-19 vaccine

## Abstract

In April 2022, a COVID-19 outbreak caused by the Omicron variant emerged in Guangzhou. A case–control study was conducted to explore the relationship between vaccination intervals and SARS-CoV-2 infection in the real world. According to the vaccination dose and age information of the cases, a 1:4 matched case–control sample was established, finally including n = 242 for the case group and n = 968 for the control group. The results indicated that among the participants who received three vaccine doses, those with an interval of more than 300 days between the receipt of the first vaccine dose and infection (or the first contact with a confirmed case) were less likely to be infected with SARS-CoV-2 than those with an interval of less than 300 days (*OR* = 0.67, 95% *CI* = 0.46–0.99). After age-stratified analysis, among participants aged 18–40 years who received two doses of vaccine, those who received the second dose more than 30 days after the first dose were less likely to be infected with SARS-CoV-2 (*OR* = 0.53, 95% *CI* = 0.30–0.96). Our findings suggest that we need to extend the interval between the first dose and the second dose and further explore the optimal interval between the first and second and between the second and third doses in order to improve vaccine efficacy.

## 1. Introduction

The situation surrounding the control of coronavirus disease 2019 (COVID-19) is still dire. The viral strain’s ongoing mutation makes it more contagious, which brings great difficulties to the prevention of the epidemic. For instance, the B.1.1.529 variant (Omicron), which is currently prevalent around the world, has traits of faster transmission and stronger immune escape [1]. It was noted that Omicron was 10-fold and 2-fold more infectious than the original severe acute respiratory syndrome coronavirus 2 (SARS-CoV-2) and Delta variant (the B.1.617.2 variant), respectively [2]. 

Vaccination has proven to be the most effective method of preventing and controlling infection with SARS-CoV-2. At present, several vaccines against COVID-19 have been developed around the world, such as inactivated vaccines, recombinant protein vaccines, adenovirus vector vaccines, and nucleic acid vaccines [3]. In clinical trials, the efficacy of these vaccines reached 95% [4,5,6]. However, research evidence suggests that variants may significantly reduce the effectiveness of COVID-19 vaccines [7]. With two doses of the ChAdOx1 vaccine, the effectiveness decreased to 74.5% for the Alpha variant and to 67.0% for the Delta variant [8]. After vaccination with BNT162b2, the geometric mean neutralization antibody titers (GMT) of the Omicron variant in serum was reduced 35.7–39.9-fold, compared with that of the ancestral virus [9]. Additionally, the results of a meta-analysis including 113 relevant studies showed that the summary effectiveness of COVID-19 vaccines to prevent Omicron infection was merely 23.5%, while the summary effectiveness for the severe diseases caused by Omicron ranged from 56.5% to 82.4%, which was lower than that of the Alpha, Beta, Gamma, and Delta variants [7].

At the same time, the titer of antibody produced by COVID-19 vaccines will gradually decline over time. A systematic review of 15,980 participants in 18 studies showed that after a second dose of BNT161b2 and Moderna, serum IgG levels peaked at 21–28 days, and thereafter the antibody titers gradually fell over the course of 4–6 months [10]. Similar findings were observed by an Israeli study [11]. With time passing by, the protective effect of the COVID-19 vaccine diminishes, and the risk of contracting SARS-CoV-2 will increase. Therefore, it is of great significance to promote boost immunization of the population. Currently, it is reported that the antibodies against the Omicron variant produced by two doses of the Pfizer vaccine are very low or undetectable, but the booster vaccination can increase it by more than 30 times [12]. Moreover, a study has shown that twelve days or more after the booster dose of the BNT162b2 vaccine, the relative risk of confirmed SARS-CoV-2 infection and severe disease will be reduced by 11.3 times and 19.5 times, respectively [13]. Consequently, booster immunization against COVID-19 not only has a better protective effect on mutant strains but also can make up for the protective effect of the vaccine that decays with time. Up to now, the boost strategy of the COVID-19 vaccines has been implemented in many countries.

Booster vaccination currently requires an interval of six months or more. However, the reality is that different people get booster shots at different time points. A study in Beijing, China, showed that during two doses of the inactivated vaccine, appropriately extending the interval between doses did not affect the production of antibodies [14]. Conversely, another research study pointed out that prolonging the vaccination interval between two doses of the BNT162b2 vaccine could enhance the production of peak antibodies [15]. Hence, there is no clear conclusion about the effect of the vaccination interval on the effectiveness of two doses of COVID-19 vaccines. 

However, in face of the currently prevalent Omicron variant, there is still a lack of relevant research on the influence of different intervals on the effectiveness of booster immunization. A new outbreak of COVID-19 related to the Omicron variant (B.1.1.529 variant) has been reported in Guangzhou since April 2022. In the recent outbreak in Guangzhou, most COVID-19 cases and their contacts received at least one dose of a COVID-19 vaccine, and some received booster shots. Compared with previous studies, this study will focus on Omicron and match cases and close contacts at a 1:4 ratio according to inoculation times and age, to evaluate the impact of their vaccination intervals on COVID-19 vaccine efficacy in a real-world setting, in this case, that of Guangzhou. Simultaneously, in the context of general booster immunization in the population, we will further concentrate on analyzing the impact of known influencing factors, such as the immunization interval between multiple vaccine doses, the time interval between the first and last doses and the onset time (for cases), and the time interval between the first and last doses and the initial contact time (for close contacts), on infection with SARS-CoV-2, thereby providing more data for better formulation of future immunization strategies.

## 2. Materials and Methods

### 2.1. Study Design and Screening of Subjects

A case–control study was conducted to assess the effect of vaccination intervals on infection with Omicron epidemic strains. In April 2022, there were 303 positive people in Guangzhou after initial nucleic acid screening. Among them, 25 cases with negative reexamination, 31 cases without vaccination (including those who received the first vaccine dose less than 14 days before infection), and 5 cases without vaccination information were excluded. Eventually, a total of 242 confirmed cases were defined as the case group.

Contacts with a high frequency and high likelihood of exposure to a SARS-CoV-2-positive case were considered as close contacts. In total, 6651 close contacts with high contact frequency were screened; of these, 1254 subjects who were not vaccinated, 294 subjects who lacked vaccination information, and 2 subjects with an unknown contact time were excluded. Finally, a total of 5101 close contacts met the inclusion and exclusion criteria.

**Figure 1:**
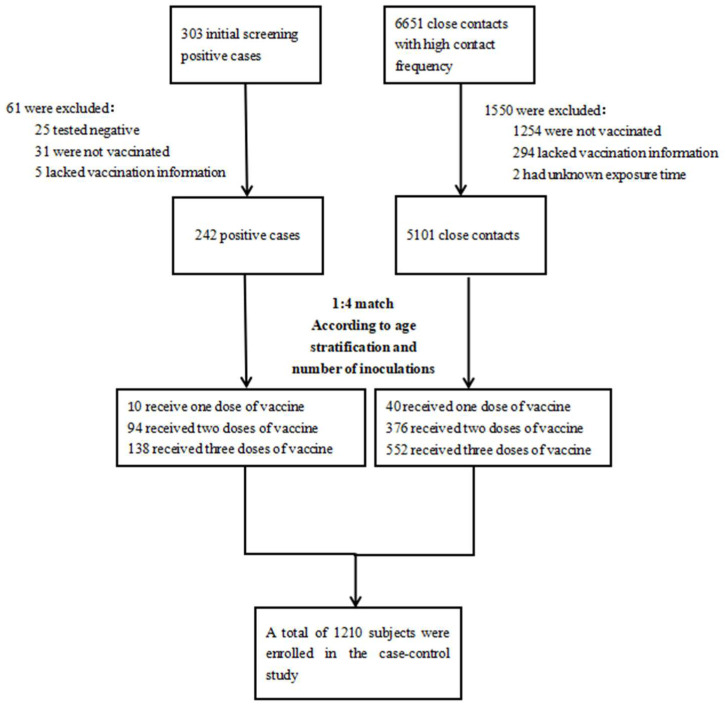
Screening process for the cases and controls.

This study selected the control group and case group at a ratio of 1:4 according to age stratification and the number of vaccine doses received. Firstly, 242 cases and 5101 close contacts were stratified according to the number of vaccination doses and age (age ≥ 18, 18 < age < 40 and age ≥ 40), and we then randomly matched the two groups to obtain the 1:4 ratio. Finally, 242 cases and 968 controls were included. The specific screening flow chart is shown in the Figure 1 below.

### 2.2. Definition of Cases and Close Contacts

In this study, a COVID-19 case was defined as a positive nucleic acid test result. Close contacts of COVID-19 refer to people who have been in close contact with COVID-19 cases but have not taken effective protective measures 2 days before the onset of symptoms, or 2 days before the sampling of asymptomatic infected persons. 

The close contacts were subjected to centralized isolation medical observation for 14 days; nucleic acid detection was performed on the 1st, 4th, 7th, and 14th days, and nasopharyngeal swabs were collected. Double sampling and double testing were performed before the isolation was lifted, and the isolation was relieved after all test results were negative (“double sampling” means that the left nasal and throat swabs were collected in one tube, and then the right nasal and throat swabs were collected in another tube, for a total of two tubes; "dual testing" means that a sample is sent to two testing facilities or tested by one facility with two different reagents).

### 2.3. Information Collection

From the close contacts, the researchers collected demographic information (age, sex, and home address); information on SARS-CoV-2 vaccination (vaccination date and type of vaccine); epidemiological information on cases (dates of positive initial screening, dates of onset, and dates and results of re-nucleic acid tests); and epidemiological information (vaccination date and type of vaccine, frequency of exposure, place of exposure, method of exposure, nucleic acid sampling date and results).

Currently, the types of COVID-19 vaccines put into use in China include inactivated vaccines, adenoviral vector vaccines, and recombinant vaccines. We collected information on the timing of vaccination and the type of vaccination, but only 4 of the 242 cases included were vaccinated with the recombinant COVID-19 vaccines. The study focused on the relationship between the length of vaccination interval and infection with the Omicron variant of SARS-CoV-2, so the type of vaccine received by the study subjects was not explained and analyzed in the subsequent data analysis.

It takes two weeks after receiving a COVID-19 vaccine to develop protection against SARS-COV-2 infection. Vaccination is considered complete if more than 14 days have passed since the first, second, or third injection to the time of positive nucleic acid test or initial contact. Otherwise, subjects were considered to have failed the last dose. For cases, it depends on whether the interval between the last dose of vaccine and the onset of illness is greater than 14 days; in turn, for close contacts, it depends on whether the interval between the last dose of vaccine and the initial exposure is greater than 14 days.

### 2.4. Stratification Criteria for Vaccination Intervals

1. TT1: The interval between the first dose of vaccine and the second dose. The second dose is generally recommended to be administered at the 21st day after the first dose. TT1 was thus classified as being greater than 30 days and less than 30 days.
2. TT2: The interval between the second dose of vaccine and the third dose. It is generally recommended to take the third dose after 180 days of the second dose, and studies have shown that the antibody level rises to the peak within one month after the third dose of vaccine, so TT2 is stratified by the 210 days.
3. TT3*: The interval between the last vaccination and infection for case group; the interval between the last injection and the first contact with confirmed cases for control group. TT3 was stratified by 60 days, 120 days, and 60 days for one-dose, two-dose, and three-dose vaccination, respectively.
4. TT4*: The interval between the first vaccination and infection for the case group; the interval between the first vaccination and the first contact of a confirmed case for the control group. The vaccination interval TT4 was stratified by 180 days for two-dose vaccination and 300 days for three-dose vaccination, respectively.
* Stratification of the TT3 and TT4 intervals: After calculating the average of the TT3 and TT4 time intervals, the average of the time intervals and the planned vaccination time were considered to determine the current stratification criteria.

### 2.5. Statistical Analysis

Statistical analysis was performed using SPSS 21.0 software (SPSS Inc., Chicago, IL, USA). Chi-square tests and T-tests were used to compare categorical variables and continuous variables, respectively. Fisher’ Exact Test was used if the classification variables do not meet the requirements of the Chi-square test; when the continuous variables did not meet the conditions, we adopted the T′-test to analyze data. Logistic regression was used to analyze the relationship between age, sex, time interval of vaccination, and SARS-CoV-2 infection. The statistical significance level was defined as *p* < 0.05. 

## 3. Results

### 3.1. Demographic Characteristics

#### 3.1.1. Demographic Characteristics of the Participants

Except for 25 cases with negative reexamination, and 5 cases without vaccination information, among the remaining 273 retested positive cases for SARS-CoV-2, 242 were vaccinated, with a higher proportion of females (55.0%), and 31 cases were unvaccinated, most being males (54.8%). However, there was no gender difference between the vaccinated and unvaccinated (*p* = 0.303). The mean age of vaccination was 33.81, with no significant difference from that of the unvaccinated group (*p* = 0.250). When stratified by age, the stratification structure of the vaccinated and unvaccinated cases differed, with the majority of vaccinated cases between the ages of 18 and 40 years (60.7%). 

The results of the statistical analysis showed that there were no statistically significant differences in age (*p* = 0.194) and gender (*p* = 0.708) between the case group and the control group (Table 1). 

#### 3.1.2. Demographic Characteristics of Cases and Controls Vaccinated with Different Doses

Likewise, between the case group and the control group with different doses, gender and age were also not significantly different (*p* > 0.05). Additionally, among the subjects who received three doses of vaccine, compared with the control group, the interval between the first or third dose of vaccine and the onset of disease in the case group was shorter (*p* = 0.001) (Table 2). 

### 3.2. Relationship between Time Interval and SARS-CoV-2 Infection

Among participants who received only one dose of a vaccine, those with a TT3 more than 60 days were likely to become infected with SARS-CoV-2 than those with a TT3 less than 60 days (*OR* = 4.85) (Table 3). However, a greater sample size is needed to confirm this result. It should be noted that stratified analyses were not performed due to the small number of cases and controls who received one dose of a vaccine. 

What is more, for participants who received two vaccine doses, there was no significant difference in SARS-CoV-2 infection among participants with different vaccination intervals. After age stratification, regarding people aged 18–40 years old, those whose TT1 intervals were more than 30 days were less likely to be infected with SARS-CoV-2 than those with TT1 intervals within 30 days (*OR* = 0.53, 95% *CI* = 0.30–0.96), which was statistically significant (*p* = 0.036). After stratifying the analysis by gender, different vaccination intervals were not statistically associated with SARS-CoV-2 infection (Table 4).

Among the participants who received three doses of a vaccine, about 66.7% of cases received a second vaccine dose within 30 days after the first dose, compared with only about 61.1% of controls, although there was no statistical difference. Those with a TT4 more than 300 days were less likely to be infected with SARS-CoV-2 than those with a TT4 less than 300 days (*OR* = 0.67, 95% *CI* = 0.46–0.99). No association was observed between SARS-CoV-2 infection and other different vaccination intervals. After adjusting for age and sex, no statistical association was found between other vaccination intervals and SARS-CoV-2 infection (Table 5).

## 4. Discussion

On 26 November 2021 [16,17], the World Health Organization (WHO) announced a new SARS-CoV-2 variant Omicron strain (B.1.1.529), which carries more abnormal mutations in its spike (S) protein than the Delta variant [18], and has multiple mutations in the receptor binding domain (RBD) of the S protein. In particular, these mutation sites (T478K, N501Y [19], D614G [20], or ΔH69/V70 [21]) on the S protein can lead to an increased affinity between SARS-CoV-2 and the ACE2 receptor, resulting in the increased transmission ability of the Omicron variant. On 8 April 2022, an outbreak of COVID-19 caused by the Omicron variant occurred in Guangzhou in the context of booster vaccination with COVID-19 vaccines. The timing of vaccination varies for each individual, and the interval of each dose may affect the risk of SARS-CoV-2 infection. Hence, it provides an opportunity for this study to explore the relationship between the vaccination intervals and SARS-CoV-2 infection, and also provides a scientific basis for the subsequent prevention and control of SARS-CoV-2. 

Among the 273 retested positive cases of SARS-CoV-2, the age distribution of the vaccinated and unvaccinated cases was different. Approximately 66.7% of the vaccinated cases were distributed between the age of 18 and 40, which may be due to the fact that at the time of COVID-19 vaccine development, the phase 1/2/3 clinical trial mainly recruited volunteers aged 18–59 [22]. Immunization was also initially carried out mainly for people aged 18–60 in China [23]. In this study, the case and control groups were matched 1:4 according to age and inoculation times. After matching, there was no significant difference in gender and age between the cases and the controls who received different doses of a vaccine. 

Regarding participants aged 18 to 40 years who received two vaccine doses, those who received the second dose 30 days and more after the first dose were less likely to be infected with SARS-CoV-2 than those who received the second dose within 30 days (*OR* = 0.53, 95% *CI* = 0.30–0.96). Through model analysis, some studies [24] have identified that 3–12 weeks is the best time for second dose vaccination. There are some real-world studies indicating that [25,26] delaying administration of the Pfizer vaccine by 12–15 weeks reduces the risk of SARS-CoV-2 infection, hospitalization, and mortality. A study [27] found that an appropriate extension of 4 weeks of vaccination can effectively reduce ICU admission rates, although these studies predate the discovery of the Omicron variant. In addition, serological and immunological studies [28] have shown that the level of S-specific B cells in the prolonged vaccination interval group is about 7 times higher than that in the control group, which can effectively prevent contracting SARS-CoV-2. However, there is still a lack of literature on whether prolonging the time interval of the third dose can effectively reduce the risk of SARS-CoV-2 infection as well.

Two doses of the vaccine cannot effectively prevent SARS-CoV-2 infection due to the transmission capacity and immune evasion of the Omicron variant, as well as the decrease in antibody level over the time of vaccination [29]. The high number of mutations in spike proteins of the Omicron variant lead to increasing infectivity and antibody evasion [18]. In essence, the COVID-19 vaccines currently in use focus on the S protein, and the 32 amino acid changes in Omicron, including three small deletions and one small insertion in the spike protein. These mutations could greatly enhance the variant’s ability to evade COVID-19 vaccines currently, rendering the currently vaccinated population unprotected [30]. Several studies [4,31] have shown that vaccine effectiveness decreases as antibody levels decline. Due to the low antibody level after one dose of a vaccine, which gradually declines over time [32], the antibody level of RBD decreased significantly 12 weeks after the second dose of the vaccine, and the antibody titer decreased by 92.46% at 7 months [33], indicating a reduction in vaccine protection. Thus, there may be no protection against Omicron variants after one or two doses of a vaccine, as the length of the interval (TT1 and TT2) between vaccination is not related to the risk of Omicron infection.

As for subjects who received three vaccine doses, the time interval from the first dose to infection or contact with a confirmed case (TT4) was higher in the controls (n = 326.28 days) than in cases (n = 309.38 days). Those with a vaccination interval (TT4) of more than 300 days were less likely to be infected with SARS-CoV-2 than those with a TT4 of less than 300 days; this might be due to the fact that about 38.9% of the participants in the control group received the second vaccine after 30 days, which was higher than that in the case group. Studies [28] have also pointed out that appropriately prolonging the time interval of the second vaccination can increase the neutralizing antibody titer and reduce the risk of SARS-CoV-2 infection. Similarly, if the second dose is administered within 30 days, the participants who received three vaccine doses may have relatively lower antibody levels than those who received the extended vaccine, thus increasing the risk of SARS-CoV-2 infection after the third booster dose. It was demonstrated [34] that higher antibody levels before the third dose are significantly associated with higher antibody levels after the booster dose. Moreover, as confirmed by another study [35], a longer interval between the second and booster dose, which means a prolonged interval of the third dose of the vaccine, was also associated with higher antibody levels after the third dose [34]. 

Our research data are mainly from Guangzhou, so they cannot completely represent the whole Guangdong province or the whole country. However, the results of this study can suggest that, in the context of the Omicron variant, we need more scientists worldwide to study antibody levels after the third dose of COVID-19 vaccines, and explore the optimal vaccination interval between the second dose and the third dose of vaccine, so as to maximize the level of antibodies after vaccination.

## 5. Conclusions

Among people aged 18 to 40 years who received two vaccine doses, those who received the second dose more than 30 days after the first dose were less likely to be infected with SARS-CoV-2. In persons who have received three vaccine doses, appropriately extending the interval between the second or third dose may reduce the risk of infection with Omicron, a variant of SARS-CoV-2. Therefore, given the global Omicron epidemic, an appropriate vaccination interval should be explored through experimental or population studies, to reduce the risk of Omicron infection.

## Figures and Tables

**Table 1:** Demographic characteristics of the 273 retested positive cases for SARS-CoV-2 and the final included cases and controls.

	Positive Cases for SARS-CoV-2				Case Group and Control Group of the Case–Control Study			
	Overall (n = 273)	Vaccinated (n = 242)	Unvaccinated (n = 31)	*p*-Value	Overall (n = 1210)	Case (n = 242)	Control (n = 968)	*p*-Value
Age				0.250				0.194
Mean (IQR)	33.24 (23.0, 43.0)	33.81 (24.0, 43.0)	28.77 (3.0, 40.0)		34.96 (25.0, 44.0)	33.81 (24.0, 43.0)	35.24 (26.0,45.0)	
Age group				0.004				1.000
Age < 18	32 (11.7%)	22 (9.1%)	10 (32.3%)		110 (9.1%)	22 (9.1%)	88(9.1%)	
18 ≤ Age ≤ 40	161 (59.0%)	147 (60.7%)	14 (45.2%)		735 (60.7%)	147 (60.7%)	588 (60.7%)	
Age > 60	80 (29.3%)	73 (30.2%)	7 (22.5%)		365 (30.2%)	73 (30.2%)	292 (30.2%)	
Gender				0.303				0.708
Male	126 (46.2%)	109 (45.0%)	17 (54.8%)		558 (46.1%)	109 (45.0%)	449 (46.4%)	
Female	147 (53.8%)	133 (55.0%)	14 (45.2%)		652 (53.9%)	133 (55.0%)	519 (53.6%)	

**Table 2:** Demographic characteristics of cases and controls vaccinated with different doses.

	1 Dose of a Vaccine			2 Doses of a Vaccine			3 Doses of a Vaccine		
	Case (n = 10)	Control (n = 40)	*p*-Value	Case (n = 94)	Control (n = 376)	*p*-Value	Case (n = 138)	Control (n = 552)	*p*-Value
Age									
Mean (IQR)	21.50 (18.8, 24.3)	27.23 (25.0, 31.0)	0.051	27.44 (18.0, 36.0)	28.76 (18.0, 36.0)	0.457	39.04 (28.0, 48.3)	40.22 (29.0, 49.8)	0.356
Gender									
Male	6 (60%)	16 (40.0%)	0.433	43 (45.7%)	190 (50.5%)	0.406	60 (43.5%)	243 (44.0%)	0.908
Female	4 (40%)	24 (60.0%)		51 (54.3%)	186 (49.5%)		78 (56.5%)	309 (56.0%)	
Time interval									
TT1(IQR)	-	-	-	33.02 (22.0, 33.0)	36.87 (23.3, 36.0)	0.315	30.02 (23.0, 32.0)	30.93 (24.0, 34.0)	0.337
TT2(IQR)	-	-	-	-	-	-	199.78 (185.8, 212.0)	201.97 (187.0, 213.0)	0.480
TT3(IQR)	216.30 (213.5, 258.8)	168.53 (34.9, 267.0)	0.126	218.05 (179.0, 259.0)	217.58 (190.0, 267.5)	0.952	79.58 (42.0, 107.0)	93.39 (53.3, 121.0)	0.001
TT4(IQR)	-	-	-	251.07 (227.5, 281.8)	254.45 (235.5, 304.0)	0.644	309.38 (274.0, 327.0)	326.28 (284.0, 355.8)	<0.001

**Table 3:** The association between vaccination intervals and SARS-CoV-2 infection among those who received one vaccine dose.

	Case	Control	*p*-Value	One dose *OR* (95% *CI*)
The whole population				
TT3 ≤ 60 days	1 (10.0%)	14 (35.0%)	Reference	-
TT3 > 60 days	9 (90.0%)	29 (65.0%)	0.153	4.85 (0.56, 42.26)

**Table 4:** The association between vaccination intervals and SARS-CoV-2 infection among those who received two vaccine doses.

	Case	Control	*p*-Value	Two Doses *OR* (95% *CI*)
The whole population				
TT1 ≤ 30 days	61 (64.9%)	212(56.4%)	Reference	-
TT1 > 30 days	33 (35.1%)	164 (43.6%)	0.136	0.70 (0.44, 1.12)
TT3 ≤ 120 days	8 (8.5%)	46 (12.2%)	Reference	-
TT3 > 120 days	86 (91.5%)	330 (87.8%)	0.314	1.50 (0.68, 3.29)
TT4 ≤ 180 days	18 (19.1%)	85 (22.6%)	Reference	-
TT4 > 180 days	76 (80.9%)	291 (77.4%)	0.469	1.23 (0.67, 2.18)
Age < 18				
TT1 ≤ 30 days	15 (71.4%)	62 (73.8%)	Reference	-
TT1 > 30 days	6 (28.6%)	22 (26.2%)	0.825	1.13 (0.39, 3.27)
TT3 ≤ 120 days	3 (14.3%)	29 (34.5%)	Reference	-
TT3 > 120 days	18 (85.7%)	55 (65.5%)	0.083	3.16 (0.86, 11.64)
TT4 ≤ 180 days	15 (71.4%)	70 (83.3%)	Reference	-
TT4 > 180 days	6 (28.6%)	14(16.7 %)	0.220	2.00 (0.66, 6.05)
18 ≤ Age ≤ 40				
TT1 ≤ 30 days	36 (62.1%)	108(46.6%)	Reference	-
TT1 > 30 days	22 (37.9%)	124 (53.4%)	0.036	0.53 (0.30, 0.96)
TT3 ≤ 120 days	5 (8.6%)	13 (5.6%)	Reference	-
TT3 > 120 days	53 (91.4%)	219 (94.4%)	0.398	0.63 (0.22, 1.84)
TT4 ≤ 180 days	3 (5.2%)	12 (5.2%)	Reference	-
TT4 > 180 days	55 (94.8%)	220 (94.8%)	1.000	1.00 (0.27, 3.67)
Age > 40				
TT1 ≤ 30 days	10 (66.7%)	42 (70.0%)	Reference	-
TT1 > 30 days	5 (33.3%)	18 (30.0%)	0.802	1.17 (0.35, 3.90)
TT3 ≤ 120 days	0 (0.0%)	4 (6.7%)	-	-
TT3 > 120 days	15 (100.0%)	56 (93.3%)	-	-
TT4 ≤ 180 days	0 (0.0%)	3 (5.0%)	-	-
TT4 > 180 days	15 (100.0%)	57 (95.0%)	-	-
Male				
TT1 ≤ 30 days	25 (58.1%)	96 (50.5%)	Reference	-
TT1 > 30 days	18 (41.9%)	94 (49.5%)	0.368	0.74 (0.38, 1.44)
TT3 ≤ 120 days	4 (9.3%)	21 (11.1%)	Reference	-
TT3 > 120 days	39 (90.7%)	169 (88.9%)	0.738	1.21 (0.39, 3.73)
TT4 ≤ 180 days	8 (18.6%)	39 (20.5%)	Reference	-
TT4 > 180 days	35 (81.4%)	151 (81.5%)	0.777	1.13 (0.49, 2.63)
Female				
TT1 ≤ 30 days	36 (70.6%)	116 (62.4%)	Reference	-
TT1 > 30 days	15 (29.4%)	70 (37.6%)	0.280	0.69 (0.35, 1.35)
TT3 ≤ 120 days	4 (7.8%)	25 (13.4%)	Reference	-
TT3 > 120 days	47 (92.2%)	161 (86.6%)	0.286	1.83 (0.61, 5.51)
TT4 ≤ 180 days	10 (19.6%)	46 (24.7%)	Reference	-
TT4 > 180 days	41 (80.4%)	140 (75.3%)	0.447	1.35 (0.63, 2.90)

**Table 5:** The association between vaccination intervals and SARS-CoV-2 infection among those who received three vaccine doses.

	Case	Control	*p*-Value	Three Doses *OR* (95% *CI*)
The whole population				
TT1 ≤ 30 days	92 (66.7%)	386 (61.1%)	Reference	-
TT1 > 30 days	46 (33.3%)	246 (38.9%)	0.221	0.79 (0.53, 1.16)
TT2 ≤ 210 days	100 (72.5%)	458 (72.5%)	Reference	-
TT2 > 210 days	38 (27.5%)	174 (27.5%)	0.999	1.00 (0.67, 1.51)
TT3 ≤ 60 days	48 (34.8%)	182 (28.8%)	Reference	-
TT3 > 60 days	90 (65.2%)	450 (71.2%)	0.165	0.79 (0.51, 1.12)
TT4 ≤ 300 days	48(34.8%)	167 (26.4%)	Reference	-
TT4 > 300 days	90 (65.2%)	465 (73.6%)	0.048	0.67 (0.46, 0.99)
18 ≤ Age ≤ 40				
TT1 ≤30 days	48 (60.0%)	233 (58.3%)	Reference	-
TT1 > 30 days	32 (40.0%)	167 (41.8%)	0.772	0.93 (0.57, 1.52)
TT2 ≤ 210 days	52 (65.0%)	285 (71.3%)	Reference	-
TT2 > 210 days	28 (35.0%)	115 (28.8%)	0.266	1.33 (0.80, 2.22)
TT3 ≤ 60 days	27 (33.8%)	104 (26.0%)	Reference	-
TT3 > 60 days	53 (66.3%)	296 (74.0%)	0.157	0.69 (0.41, 1.15)
TT4 ≤ 300 days	21 (26.3%)	85 (21.3%)	Reference	-
TT4 > 300 days	59 (73.8%)	315 (78.8%)	0.326	0.76 (0.44, 1.32)
Age > 40				
TT1 ≤ 30 days	44 (75.9%)	153 (65.9%)	Reference	-
TT1 > 30 days	14 (24.1%)	79 (34.1%)	0.150	0.62 (0.32, 1.19)
TT2 ≤ 210 days	48 (82.8%)	173 (74.6%)	Reference	-
TT2 > 210 days	10 (17.2%)	59 (25.4%)	0.193	0.61 (0.29, 1.28)
TT3 ≤ 60 days	21 (36.2%)	78 (33.6%)	Reference	-
TT3 > 60 days	37 (63.8%)	154 (66.4%)	0.710	0.89 (0.50, 1.63)
TT4 ≤ 300 days	27 (46.6%)	82 (35.3%)	Reference	-
TT4 > 300 days	31 (53.4%)	150 (64.7%)	0.117	0.62 (0.35, 1.12)
Male				
TT1 ≤ 30 days	38 (63.3%)	164 (55.6%)	Reference	-
TT1 > 30 days	22 (36.7%)	131 (44.4%)	0.271	0.73 (0.41, 1.29)
TT2 ≤ 210 days	48 (80.0%)	216 (73.2%)	Reference	-
TT2 > 210 days	12 (20.0%)	79 (26.8%)	0.275	0.68 (0.35, 1.35)
TT3 ≤ 60 days	19 (31.7%)	79 (26.8%)	Reference	-
TT3 > 60 days	41 (68.3%)	216 (73.2%)	0.441	0.80 (0.43, 1.44)
TT4 ≤ 300 days	21 (35.0%)	77 (26.1%)	Reference	-
TT4 > 300 days	39 (65.0%)	218 (73.9%)	0.162	0.66 (0.36, 1.18)
Female				
TT1 ≤ 30 days	54 (69.2%)	222 (65.9%)	Reference	-
TT1 > 30 days	24 (30.8%)	115 (34.1%)	0.572	0.86 (0.51, 1.50)
TT2 ≤ 210 days	52 (66.7%)	242 (71.8%)	Reference	-
TT2 > 210 days	26 (33.3%)	95 (28.2%)	0.368	1.27 (0.75, 2.16)
TT3 ≤ 60 days	29 (37.2%)	103 (30.6%)	Reference	-
TT3 > 60 days	49 (62.8%)	234 (69.4%)	0.259	0.74 (0.45, 1.24)
TT4 ≤ 300 days	27 (34.6%)	90 (26.7%)	Reference	-
TT4 > 300 days	51 (65.4%)	247 (73.3%)	0.163	0.69 (0.41, 1.16)

## Data Availability

Not applicable.
